# Acute Pancreatitis Caused by Impaction of Migrated Surgical Staples in the Duodenal Papilla After Distal Pancreatectomy

**DOI:** 10.1002/deo2.70465

**Published:** 2026-11-03

**Authors:** Kazumi Inaba, Mitsuteru Motomura, Yoshifumi Azuma, Takahiro Kimoto, Haruhiko Takahashi, Tetsuya Ueo, Ryoichi Narita, Kazuhiro Tada, Kengo Fukuzawa, Kazuhiro Mizukami

**Affiliations:** ^1^ Department of Gastroenterology Oita Red Cross Hospital Oita Japan; ^2^ Department of Hepatobiliary and Pancreatic Disease Oita Red Cross Hospital Oita Japan; ^3^ Department of Surgery Oita Red Cross Hospital Oita Japan; ^4^ Department of Gastroenterology, Faculty of Medicine Oita University Oita Japan

**Keywords:** acute pancreatitis, endoscopic retrograde cholangiopancreatography, foreign‐body migration, pancreatectomy, surgical staplers

## Abstract

A 67‐year‐old woman underwent a laparoscopic distal pancreatectomy for pancreatic body cancer. On postoperative day (POD) 23, the patient presented with epigastric pain and was diagnosed with delayed peripancreatic fluid collection, which was treated with antibiotics and percutaneous drainage. On POD 51, the patient developed recurrent epigastric pain. Laboratory tests revealed elevated pancreatic enzyme and inflammatory marker levels. Computed tomography revealed acute pancreatitis and a high‐density area in the duodenal papilla suggestive of a foreign body. Endoscopic retrograde cholangiopancreatography revealed that the surgical staples were impacted in the minor duodenal papilla and were successfully removed. Although pancreatography did not demonstrate residual staples, three additional staples were subsequently removed from the pancreatic duct. Pancreatitis improved after endoscopic treatment, and the patient was discharged. This case suggests that the migration of staples used during surgery may be a contributing factor to postoperative pancreatitis.

## 1 Introduction

Acute pancreatitis is commonly caused by alcohol consumption and gallstones, whereas foreign‐body pancreatitis is extremely rare [1]. Also, common complications after pancreatic resection include peripancreatic fluid collection (PFC), postoperative bleeding, and delayed gastric emptying, whereas acute pancreatitis is uncommon [2]. Reported risk factors for postoperative acute pancreatitis include female sex, high BMI, minimal pancreatic fibrosis, and a narrow main pancreatic duct [3]. Postoperative acute pancreatitis can become severe and warrants careful attention.

Postoperative foreign‐body pancreatitis or cholangitis has been reported sporadically and is most often caused by migration of surgical clips into the bile duct after hepatobiliary surgery [4]. To our knowledge, foreign‐body pancreatitis after pancreatic resection is extremely rare, with only one case previously reported [5].

We report a rare case of acute pancreatitis caused by impaction of migrated surgical staples at the minor duodenal papilla after pancreatic resection, which was successfully treated with endoscopic retrograde cholangiopancreatography (ERCP).

## 2 Case Report

A 67‐year‐old woman was diagnosed with an intraductal papillary mucinous neoplasm (IPMN) in the pancreatic tail during a health checkup 2 years ago. During routine follow‐up examinations, computed tomography (CT) and magnetic resonance imaging (MRI) revealed an 8 mm mass in the pancreatic body. The patient was then referred to our surgical department. Her medical history included dyslipidemia.

Contrast CT revealed an 8 mm low‐density lesion in the pancreatic body, and contrast enhancement was observed in the arterial phase, which gradually decreased in the subsequent phases (Figure 1a,b). On diffusion‐weighted MRI, the lesion showed a slightly high signal, but without a decline in ADL (Figure 1c). MRI revealed a main pancreatic duct (MPD) stricture in the pancreatic body, with upstream dilation. The previously noted IPMN was observed in the pancreatic tail. Although the MPD communicated with the accessory pancreatic duct (APD), the APD was wider than the MPD, indicating an incomplete type of pancreas divisum (Figure 1d).

**FIGURE 1:**
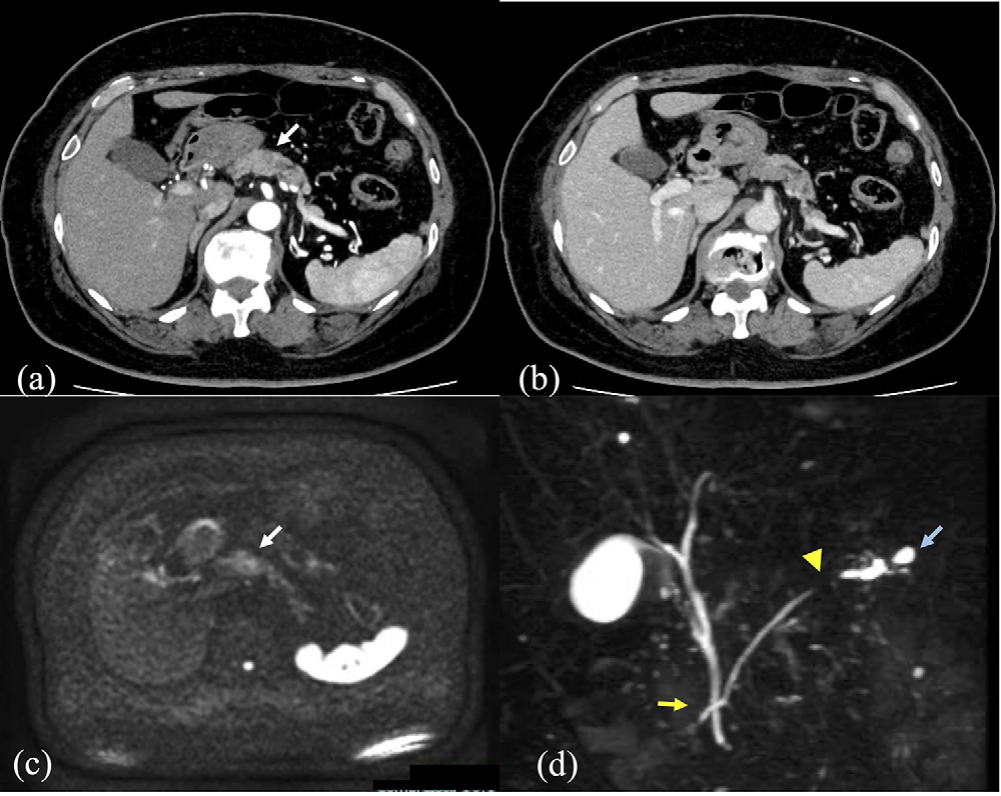
Computed tomography (CT) and Magnetic resonance imaging (MRI) findings. (a) Contrast CT revealed an 8 mm low‐density lesion in the pancreatic body, and contrast enhancement was observed in the arterial phase. (b) Contrast enhancement was observed gradually decreased in the subsequent phase. (c) On diffusion‐weighted MRI, the lesion showed a slightly high signal. (d) MRI revealed a main pancreatic duct (MPD) stricture in the pancreatic body, with upstream dilation (head arrow). The previously noted IPMN was observed in the pancreatic tail (blue arrow). The accessory pancreatic duct was wider than the MPD, indicating an incomplete type of pancreas divisum (yellow arrow).

Endoscopic ultrasound (EUS) revealed a 12 mm irregular hypoechoic mass in the pancreatic body, and the distal MPD was dilated (Figure 2). Based on this finding, we suspected pancreatic cancer.

**FIGURE 2:**
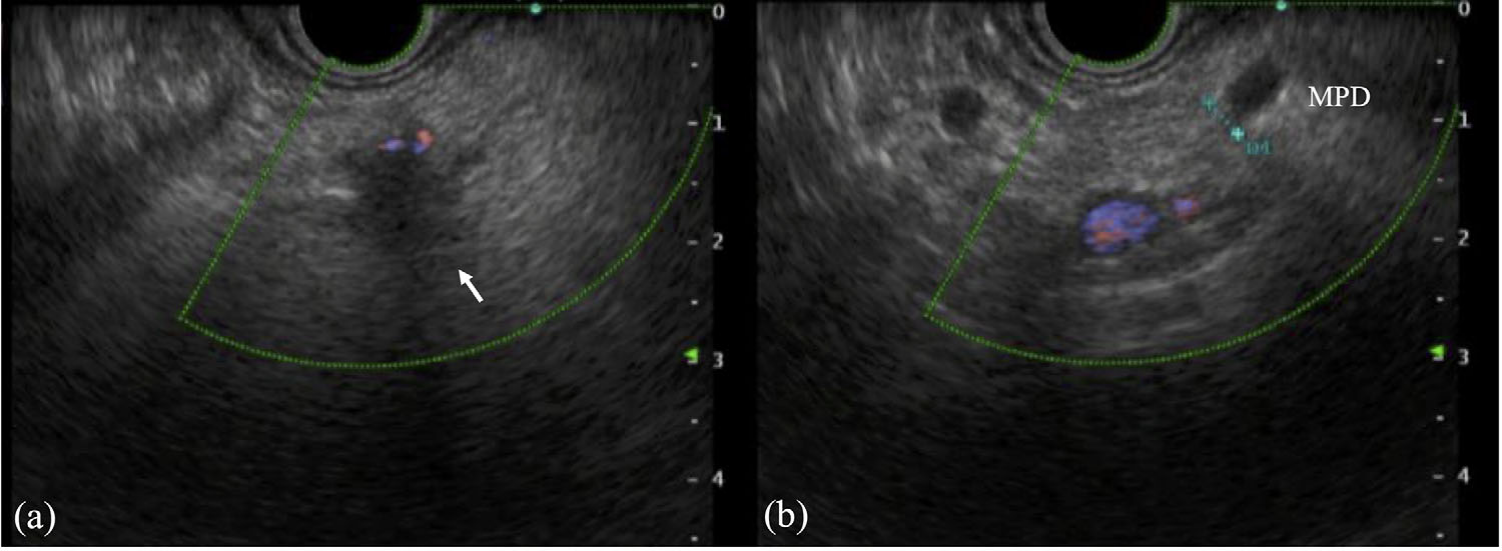
Endoscopic ultrasonography (EUS) findings. (a) A 12 mm irregular hypoechoic mass was observed in the pancreatic body (arrow). (b) The main pancreatic duct (MPD) distal to the tumor was dilated.

Regarding ERCP, cannulation to the minor duodenal papilla can be difficult; furthermore, given the narrow MPD and the limited drainage pathways compared to normal anatomy, the risk of post‐ERCP pancreatitis and a subsequent treatment delay was considered. Although uncommon, EUS‐guided tissue acquisition included the risk of seeding along the needle tract. We considered that the treatment plan would not change even if these examinations were negative. Therefore, treatment was initiated after obtaining informed consent.

GS therapy (gemcitabine + S‐1 [tegafur, gimeracil, and oteracil potassium]) was administered as neoadjuvant chemotherapy because of anterior infiltration. However, chemotherapy was discontinued after one course of the drug rush.

Three months after the diagnosis, laparoscopic distal pancreatectomy was performed. The surgery lasted for 4 h and 20 min, and blood loss was 280 mL. The pancreas was resected immediately above the portal vein using an automatic stapler (Powered ECHELON FLEX 3000; Johnson&Johnson MedTech, USA). Histopathological examination revealed a well‐differentiated adenocarcinoma with no lymph node metastasis and negative resection margins. The pathological TNM stage was pT3N0M0 (Stage IIA). The postoperative course was uneventful, and the patient was discharged on postoperative day (POD) 9.

On POD 23, the patient complained of epigastric pain. CT revealed a fluid collection around the pancreatic resection margin; thus, we diagnosed a PFC (Figure 3a). Antibiotic therapy did not improve the PFC. As the PFC was located close to the abdominal wall, percutaneous drainage was performed on POD 34. Then the PFC improved, and the drain was clamped on POD 47. The patient complained again of epigastric pain on POD 51. Laboratory tests revealed elevated pancreatic enzyme and inflammatory marker levels. CT revealed pancreatic enlargement and increased density of the surrounding adipose tissue, suggesting acute pancreatitis. A high‐density area was identified in the duodenal papilla, which was considered the cause of pancreatitis (Figure 3b). CT findings on POD 23 and 51 revealed staples at the pancreatic resection margin, with no clear differences in their number or position (Figure 3c,d). The patient was managed conservatively and underwent ERCP on POD 52. ERCP revealed surgical staples impacted in the minor duodenal papilla (Figure 4a). No foreign objects were identified on the X‐ray. A precut was performed using a needle scalpel, and the three staples were removed using biopsy forceps (Figure 4b). Pancreatography did not identify any staples. Endoscopic pancreatic sphincterotomy was performed (Clever Cut 3 V, 3 mm; Olympus, Japan), and balloon catheter sweeping of the pancreatic duct (Extraction balloon catheter ProXL; Zeon Medical, Japan) removed three additional staples (Figure 4c,d). The procedure was completed by placing a pancreatic stent (Geenen, 5 Fr × 5 cm; Cook Medical, Japan). Subsequently, the pancreatitis improved. Three months later, the pancreatic stent was removed via ERCP.

**FIGURE 3:**
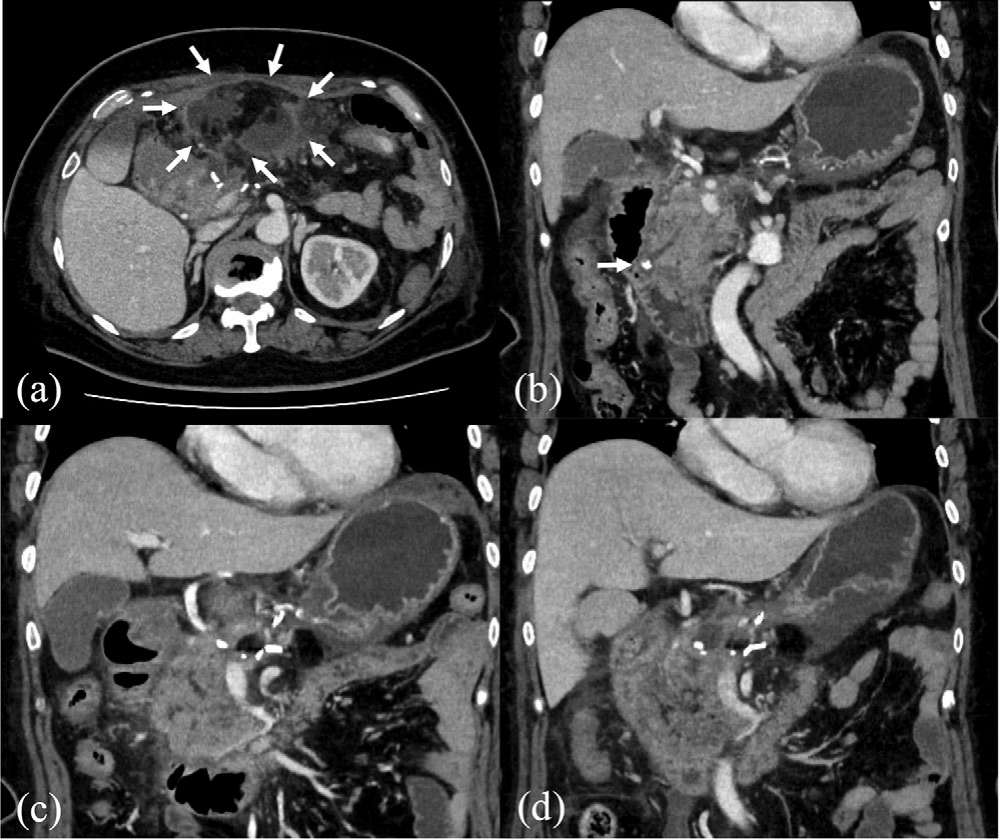
Computed tomography (CT) findings. (a) Encapsulated fluid was observed from the pancreatic stump to the epigastric region. (b) The pancreas was enlarged, suggesting acute pancreatitis, and a high‐density area was observed in the duodenal papilla with dilatation of the distal pancreatic duct. (c, d) CT findings on POD 23 and 51 revealed staples at the pancreatic resection margin; no clear differences in their number or position were observed.

**FIGURE 4:**
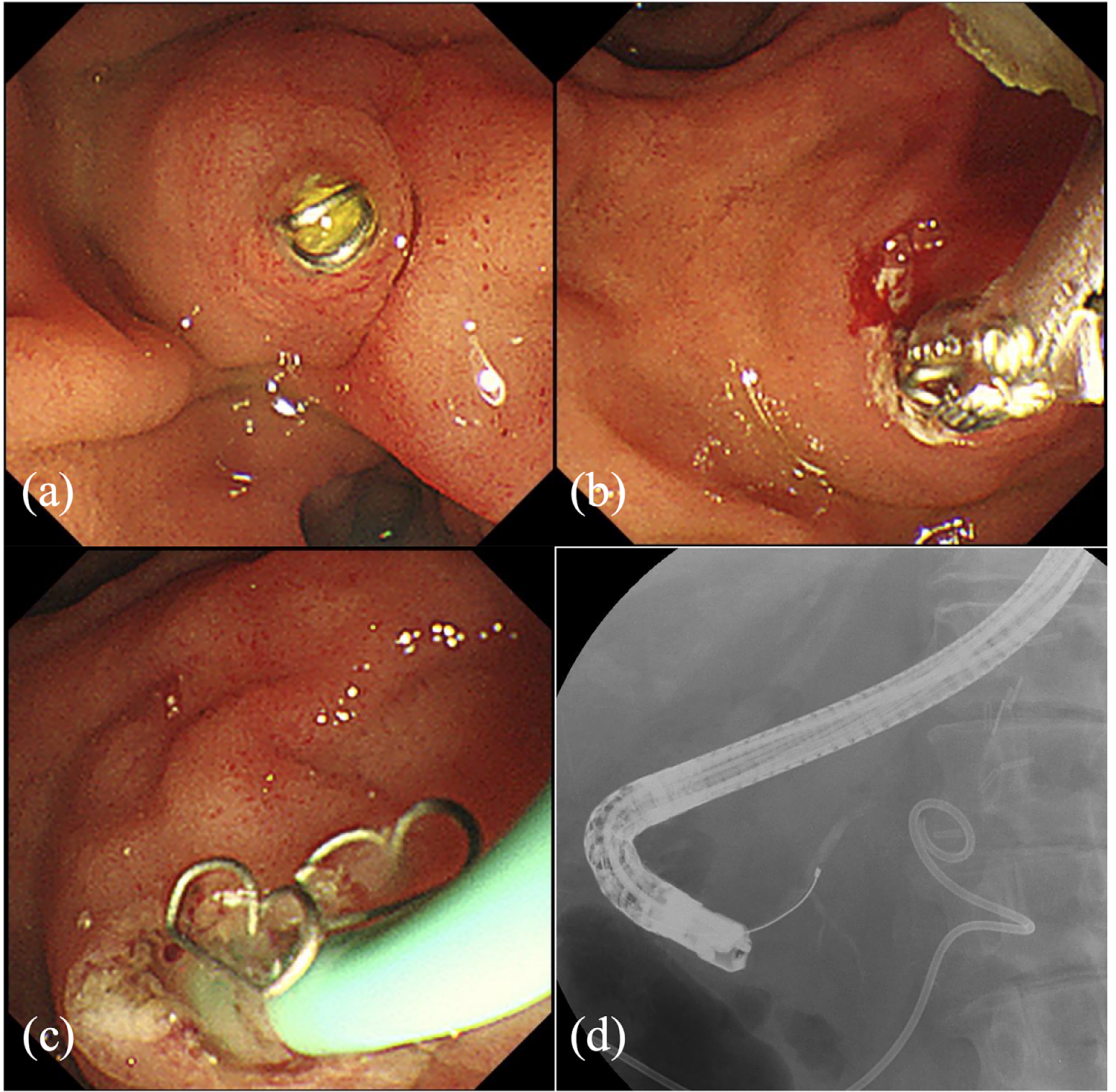
Endoscopic retrograde cholangiopancreatography findings. (a) The staples impacted in the minor duodenal papilla. (b) The staples were removed using biopsy forceps. (c) Additional staples were removed using a balloon catheter. (d) Pancreatography did not reveal any staples.

## 3 Discussion

In the present case, surgical staples migrated into the pancreatic duct after pancreatic resection and became impacted in the minor duodenal papilla, resulting in acute pancreatitis. To our knowledge, this is the first case of staple impaction in the minor duodenal papilla confirmed by both CT and endoscopy.

The complications of pancreatic resection include PFC, postoperative bleeding, and delayed gastric emptying [6]. PFC is relatively common, occurring in 5%–30% of cases after pancreaticoduodenectomy and 20%–31% of cases after distal pancreatectomy. Chui et al. suggested that acinar cell damage in the remnant pancreas plays a central role, with local inflammation, ischemia, surgical manipulation, and mechanical stress contributing to the development of PFC [7]. Although the reported incidence of postoperative acute pancreatitis is only 3.6% [2], it remains clinically important because pancreatic resection is highly invasive, and this complication may affect prognosis.

Postoperative foreign‐body migration has been reported sporadically as cholangitis or pancreatitis caused by clip migration after hepatobiliary surgery. Kihara et al. reported a 0.5% incidence of postoperative clip migration into the bile duct [8]. Although the mechanism remains unclear, reported explanations for clip migration include tissue necrosis caused by inversion of the cystic duct into the bile duct due to compression by the surrounding tissue, tissue necrosis due to bile leakage, and incomplete closure of the cystic duct [9]. These findings suggest that changes in the local postoperative environment may contribute to foreign‐body migration.

On the other hand, foreign‐body migration into the pancreatic duct after pancreatic resection has rarely been reported [5, 10]. Similar to the present case, postoperative PFC was observed in the case reported by Soga et al. [10]. Both reports suggested that postoperative pancreatic fluid collections and inflammation or tissue damage may have contributed to staple or clip migration [5, 10].

In the present case, the patient also had prolonged PFC, and inflammation and tissue fragility may have contributed to staple migration. In addition, the drain was clamped four days before the diagnosis of acute pancreatitis; thus, changes in the pressure of the pancreatic leak cavity and pancreatic duct may also have contributed to the staple migration. Because the staples were within the MPD, we assumed that the staples from the pancreatic resection margin migrated through the MPD to the APD and became impacted at the minor duodenal papilla. The patient had incomplete pancreas divisum, indicating that the APD served as the primary drainage pathway for the pancreatic juice. Therefore, we thought that obstruction of the minor duodenal papilla with the impacted staples had caused acute pancreatitis. No staples were observed within the MPD on the CT scan on POD 23; the timing of their migration remains unknown.

The staples were not detected on the X‐ray or pancreatography; however, residual staples were removed from the pancreatic duct by balloon catheter sweeping. Therefore, the absence of a visible foreign body on pancreatography does not exclude their presence in the pancreatic duct. If foreign‐body obstruction is clinically suspected, active exploration of the pancreatic duct should be considered. Endoscopic foreign‐body removal and pancreatic duct stenting resulted in a favorable outcome.

We have experienced an extremely rare case of acute pancreatitis caused by surgical staple migration into the pancreatic duct, with subsequent impaction in the minor duodenal papilla after pancreatic resection. When acute pancreatitis of unknown etiology occurs after pancreatic resection, mechanical obstruction due to the migration of a foreign body should also be considered in the differential diagnosis.

## Author Contributions

**Kazumi Inaba**: conceptualization, investigation, and writing of the original draft. **Mitsuteru Motomura**: supervision, writing, review, and editing. **Yoshifumi Azuma**: writing, review, and editing. **Takahiro Kimoto**: writing, review, and editing. **Haruhiko Takahashi**: writing, review, and editing. **Tetsuya Ueo**: supervision, writing, review, and editing. **Ryoichi Narita**: supervision, writing, review, and editing. **Kazuhiro Tada**: writing, review, and editing. **Kengo Fukuzawa**: supervision and conceptualization. **Kazuhiro Mizukami**: supervision and conceptualization.

## Funding

The authors have nothing to report.

## Ethics Statement

Institutional Review Board approval was not required for this case report in accordance with the local guidelines.

## Consent

Informed consent for publication was obtained from the patient.

## Conflicts of Interest

The authors declare no conflicts of interest.

## Data Availability

The data that support the findings of this study are available on request from the corresponding author. The data are not publicly available due to privacy or ethical restrictions.
